# The complete mitochondrial genome of *Sinularia humilis* van Ofwegen, 2008 (Octocorallia: Alcyonacea)

**DOI:** 10.1080/23802359.2022.2095232

**Published:** 2022-07-19

**Authors:** Chaojie Yang, Zhongjie Wu, Yujing Huang

**Affiliations:** a Key Laboratory of Utilization and Conservation for Tropical Marine Bioresources (Hainan Tropical Ocean University), Ministry of Education, Sanya, China; b Hainan Academy of Ocean and Fisheries Sciences, Haikou, China

**Keywords:** Mitogenome, soft coral, *Sinularia humilis*, phylogenetic

## Abstract

In this study, the complete mitochondrial genome (mitogenome) of *Sinularia humilis* van Ofwegen, 2008 was determined using next-generation sequencing (NGS) method. The mitogenome of *S. humilis* is 18,743 bp in length, containing 14 protein-coding genes (PCGs), two ribosomal RNAs (rRNA) and one tRNA (tRNA-Met), which has same gene order with other species of *Sinularia.* ATG was determined as start codon in all 14 PCGs. Eight TAG, five TAA, and one incomplete codons (T-) were found as stop codon. Phylogenetic analysis of the small number of available mitogenomes showed that *S. humilis* is closely related to *Sinularia ceramensis* and *Sinularia peculiaris*.

The genus *Sinularia* with approximately 170–190 biological species is a large group in Octocorallia, commonly distributed in tropical zones of shallow water to deep reefs (van Ofwegen 2000; Fabricius and Alderslade 2001; McFadden et al. 2009). van Ofwegen has described species of *Sinularia humilis* from Palau (Micronesia) in 2008. Further studies have documented distribution of this species in South China Sea (Benayahu et al. 2012, 2018).

So far, only six mitogenomes of genus *Sinularia* have been sequenced (Asem et al. 2019; Chen et al. 2019; Shen et al. 2021); their mitogenome lengths ranged from 18,730 to 18,742 bp. In the present study, complete mitochondrial genome of *S. humilis* (GenBank no. OK641586) was sequenced and annotated to study the mitogenomic characteristics and its phylogenetic relationships within *Sinularia*.

With regard to regulations of Department of Science and Technology of Hainan province government, a permission was obtained to collect a sample from China South Sea following R&D program under ZDKJ2019011-03-02 reference number. A specimen of S. *humilis* was collected from the South China Sea (Small Island, Sanya, China; 18°15′12″N-109°30′13″E) and stored in Hainan Tropical Ocean University Museum of Zoology (specimen voucher: HTOU-SiHu001; Chaojie Yang; duanduan1986@outlook.com). The specimen was identified based on morphology of sclerites (Benayahu, personal communication). Total genomic DNA was extracted with Genomic DNA Isolation Kit no. B518221 (Sangon Biotech Co., Shanghai, China) (Asem et al. 2021).

The quality of extracted DNA was checked on a 1.5% agarose gel and then quantified using a Micro-volume Spectrophotometer (MaestroGen Inc., Hsinchu City, Taiwan). An amount of 600 ng of total DNA was used to construct the genomic library with paired-end (2 × 150 bp) followed by next-generation sequencing (NGS) (10 Gb) approach. The sequencing was performed on the Illumina HiSeq X-ten sequencing platform (Novogene Co., Tianjin, China). Total sequencing included effective rate, 0.03% of error rate and 90.31% of Q30. Quality of reads were controlled by FastQC. De novo assemblies were performed with Geneious 9.1 utilizing the mitogenome of *Sinularia ceramensis* (GenBank no. NC_044122) as reference map with default parameter settings (Kearse et al. 2012). The position of tRNA-Met gene was determined by ARWEN software (http://130.235.46.10/ARWEN/). PCGs and rRNAs were annotated by multiple sequence alignments to the reference mitogenome using BioEdit program (Hall 1999). In addition, all PCGs were translated into amino acids by the ExPASy online program (https://web.expasy.org/translate/) and sequences were examined to ensure that each could encode a functional protein.

The length of mitogenome of S. *humilis* was 18,743 bp including 14 protein-coding genes (PCGs: 14,804 bp), two ribosomal RNAs (rRNAs: 3260 bp), and one transfer RNA (tRNA-Met: 17 bp). The overall nucleotide composition of the heavy strand was as follows: 30.35% A, 16.52% C, 19.29% G, and 33.83% T, with a total A + T content of 64.18%. Four PCGs including COX3, ATP6, ATP8, and COX2 and tRNA-Met were located on the light strand. All PCGs encoded with common ATG start codon. Stop codons included eight TAG (ND1, CYTB, ND6, ND3, ND2, ND5, COXIII, and COXII), five TAA (ND4L, MutS, ND4, ATP6, and ATP8), and a non-complete codon T (COXI). The 12S rRNA and 16S rRNA were encoded on the heavy strand from 1589 to 2634 (1046 bp) and 9159 to 11,369 (2211 bp), respectively. Same as other *Sinularia* mitogenome, 16S rRNA showed a rather higher A + T content (58.07% vs. 56.69%) in S. *humilis*. The longest overlap and gap were observed between ND2/ND5 with 13 bp and COX2/COX1 with 112 bp, respectively.

The phylogenetic relationship of S. *humilis* among other species of *Sinularia* was inferred with a concatenated dataset including the 14 PCGs and two rRNAs using maximum-likelihood (ML). *Sarcophyton trocheliophorum* (MK994517) was used as an outgroup (Shen et al. 2019). The ML phylogenetic analysis was performed using MEGA X. The best-fitting nucleotide substitution model was calculated based on the results of the MrModelTest 2.2 (Alfaro et al. 2003) and HKY + G was chosen as the best-fit model with 1000 bootstrap replicates (Figure 1). Regarding available mitogenomes, results revealed that *Sinularia* was divided in two clades and that *S. humilis* was closely related to *Sinularia ceramensis* and *Sinularia peculiaris*. Further studies with large number of *Sinularia* mitogenomes need to represent clear status of species of genus *Sinularia*.

**Figure 1.**
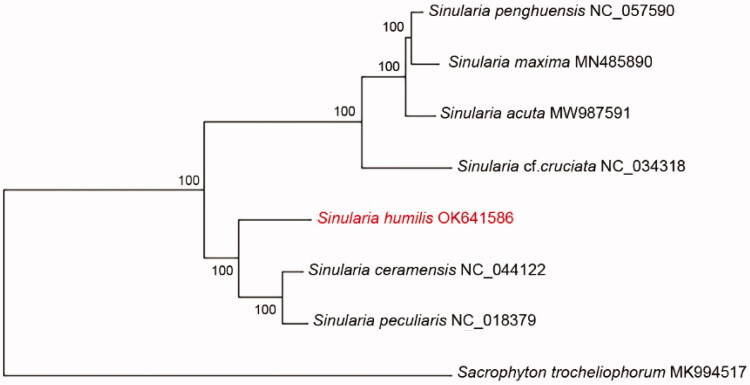
Phylogenetic tree shows evolutionary relationships among genus *Sinularia* based on maximum-likelihood (ML) approach. Numbers adjacent to nodes denote the bootstrap support values. The GenBank accession numbers are indicated on the right side of species names.

## Data Availability

The data that support the findings of this study are openly available in GenBank of NCBI at https://www.ncbi.nlm.nih.gov, reference number OK641586. The associated BioProject, Bio-Sample, and SRA numbers are PRJNA780662, SAMN23175337, and SRR16960927, respectively.
